# Supplementary material for: Cross-stage neural pattern similarity in the hippocampus predicts false memory derived from post-event inaccurate information
Source: Nat Commun. 2023 Apr 21;14:2299. doi: 10.1038/s41467-023-38046-y (PMC10121656; doi:10.1038/s41467-023-38046-y)
Supplement: Supplementary file 1 — Supplementary Information [file 41467_2023_38046_MOESM1_ESM.pdf]

**Supplementary Information for**

# Cross-stage neural pattern similarity in the hippocampus predicts false memory derived from post-event inaccurate information

Xuhao Shao, Ao Li, Chuansheng Chen, Elizabeth F. Loftus, Bi Zhu*

*Correspondence author:

Bi Zhu,

Email: zhubi@bnu.edu.cn

This PDF file includes:

Supplementary Figures 1 to 11

Supplementary Tables 1 to 17

Supplementary Methods

Supplementary References

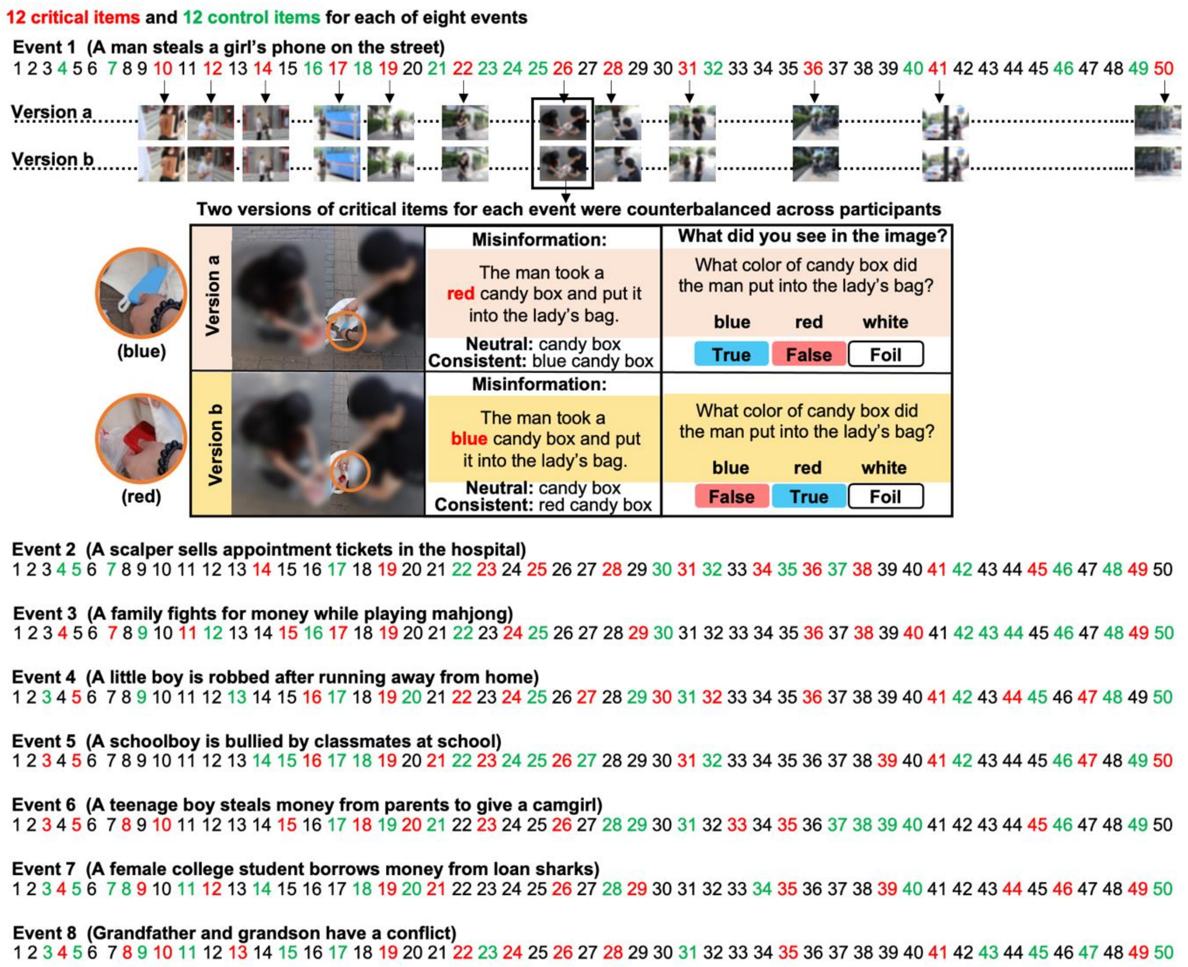


**Supplementary Figure 1. Materials of eight original events.** For each of eight events, 50 images were used to describe one theme, including 12 critical items (colored in red) and 12 control items (colored in green) that would be tested later. To increase the credibility of narratives, critical and control items would not be set to the first two slides of each event, and any two of critical items from each event would not appear one after the other (i.e., they would be interspersed with generic and control items). To obtain a balanced design, two versions of critical items were developed and counterbalanced across participants. For example, in the misinformation group, one participant saw an image depicted that a man took a blue candy box, and later would read the misinformation of a red candy box; whereas another participant saw an alternative image depicted that a man took a red candy box, and later would read the misinformation of a blue candy box. Images of people in the figure are blurred according to journal's regulations, while images in the experiment were displayed in high resolution. Permission has been obtained from these individuals to depict them in this figure.

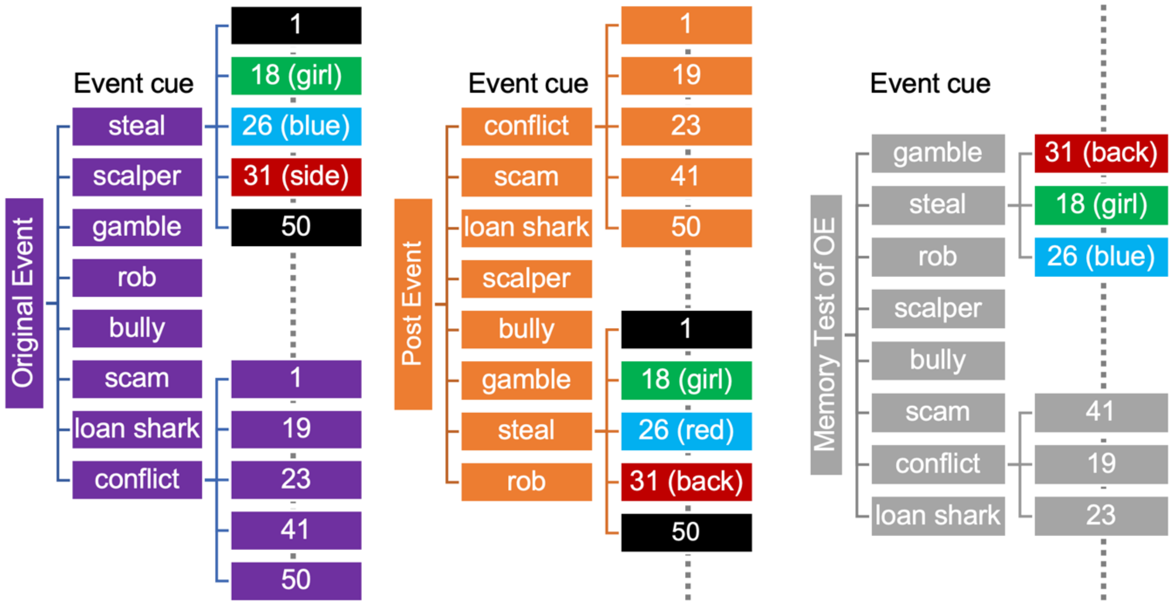


**Supplementary Figure 2. Schematic display of the randomized presentation order for critical (blue and red for true and false memories, respectively), control (green for correct control), and generic (black for untested) items from eight events at the three stages of original-event (purple), post-event (orange), and memory-test (grey).**

As shown in the color of blue, an example for true memory is described as follows. A participant saw the **26$^{th}$** of 50 images (i.e., *the man took a **blue** candy box and put it into the lady's bag*) from the 1$^{st}$ presented original event about stealing. Then, the participant read the corresponding misinformation (i.e., *the man took a **red** candy box and put it into the lady's bag*) from the 7$^{th}$ presented narrative about stealing. Finally, the participant chose the original option of "**blue**", when asked about the 3$^{rd}$ presented question (i.e., *What color of candy box did the man put into the lady's bag?*) during the memory test about stealing.

As shown in the color of red, an example for false memory is described as follows. A participant saw the **31$^{st}$** of 50 images from the original event about stealing (i.e., *the man put the stolen phone in his pants' **side** pocket*). Then, the participant read the post-event misinformation (i.e., *the man put the stolen phone in his pants' **back** pocket*). Finally, the participant chose the misinformation option of "***back*** pocket", when asked about the 1$^{st}$ presented question (i.e., *Which pocket of his pants did the man put the stolen phone in?*) during the memory test.

As shown in the color of green, an example for correct control is described as follows. A participant saw the **18$^{th}$** of 50 images from the original event about stealing (i.e., *the man was crossing the street, next to an old lady and a little **girl***). Then, the participant read the post-event consistent information (i.e., *the man was crossing the street, next to an old lady and a little **girl***). Finally, the participant chose the correct option of "***girl***" when asked about the 2$^{nd}$ presented question (i.e., *When the man was crossing the street, who was walking with an old lady?*) during the memory test.

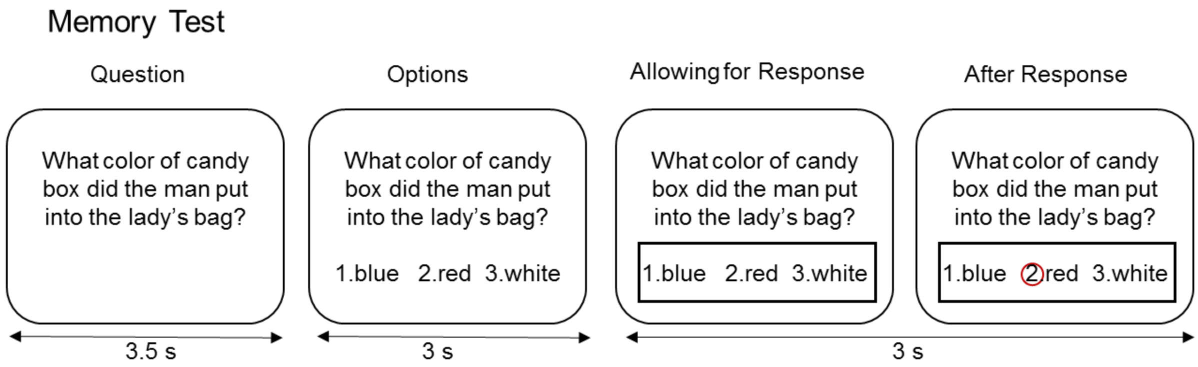


**Supplementary Figure 3. Experimental procedure for each trial in the memory test.**

s = seconds.

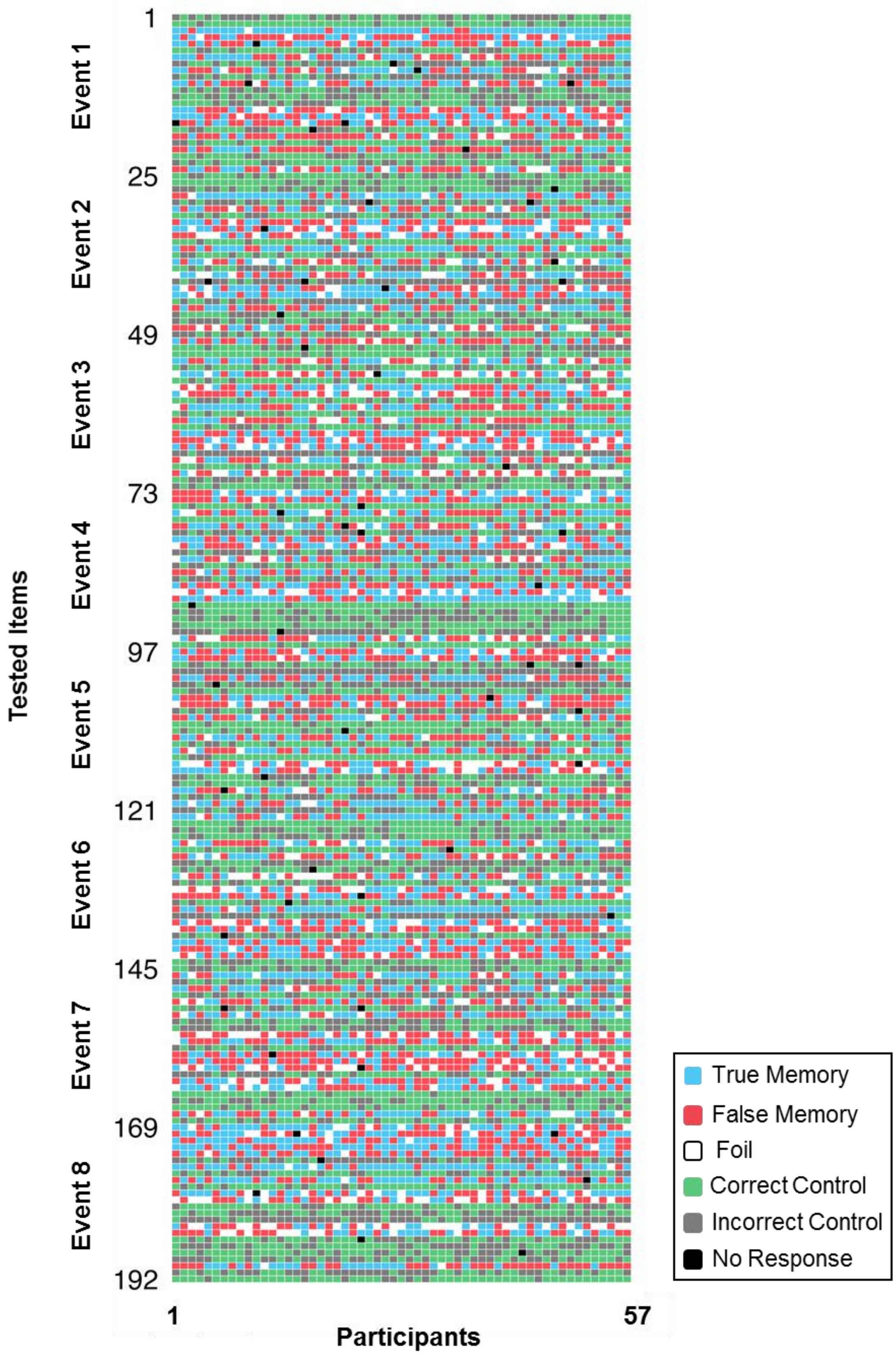


**Supplementary Figure 4. Memory responses for 192 tested items in 57 participants of Exp. 2.**

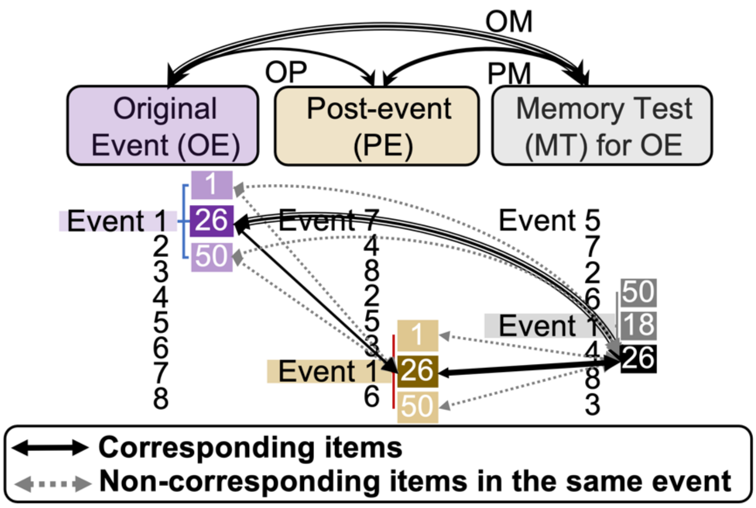


**Supplementary Figure 5. Item-specific representation was indicated by greater neural pattern similarity across stages for the corresponding items (i.e., black solid line) than that for the non-corresponding items in the same event (i.e., grey dashed line).** Across three stages, there are three types of similarity (i.e., OP [between original-event and post-event stages], PM [between post-event and memory-test stages], and OM [between original-event and memory-test stages]). For example, one participant saw the image depicting "the man took a blue candy box" during the original-event stage (image 26), and then read the misinformation "the man took a red candy box" during the post-event stage (narrative 26). If this participant retrieved the original information (i.e., blue candy box) while reading the misinformation, then this participant was more likely to produce a true memory than a false memory in the subsequent memory test. In this case, the cross-stage neural pattern similarity between the original-event and post-event stages was caused by the overlap between the encoding of the original information during the original-event stage (i.e., the man took a blue candy box) and the retrieval of the original information during the post-event stage (i.e., the man took a blue candy box).

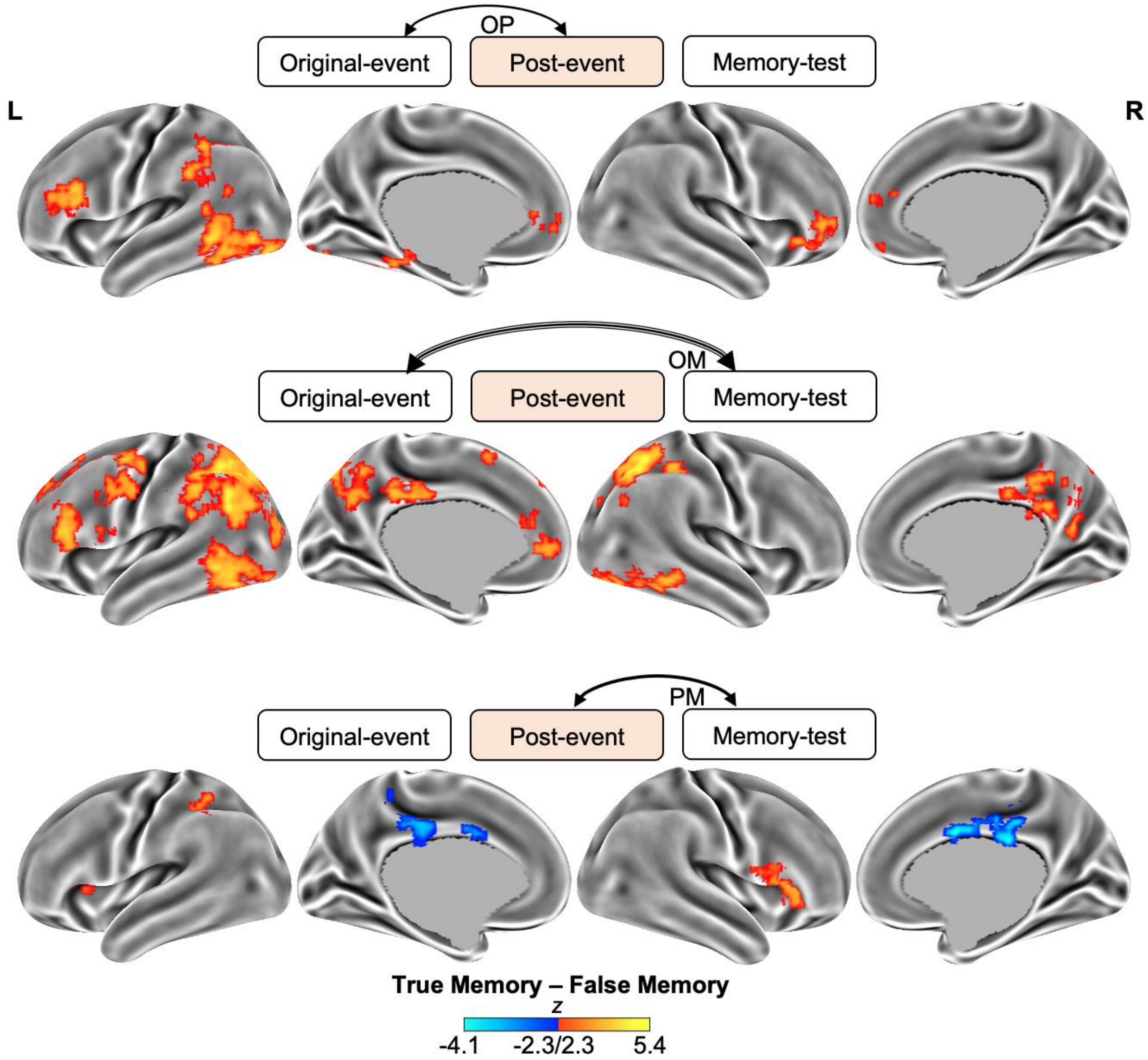


**Supplementary Figure 6. Brain regions showing differences between true and false memories in item-specific neural pattern similarity for OP (between original-event and post-event stages), OM (between original-event and memory-test stages), and PM (between post-event and memory-test stages) in Exp. 2 (thresholded at Z > 2.3).** Multiple brain regions showed greater item-specific neural pattern similarity between the original-event and post-event stages for true memory than false memory, whereas no brain region showed the reversed pattern. These results suggest that the cross-stage neural pattern similarity between the original-event and post-event stages is mainly due to the retrieval of original information during the post-event stage, rather than due to the blurring of the two pieces of encoded information.

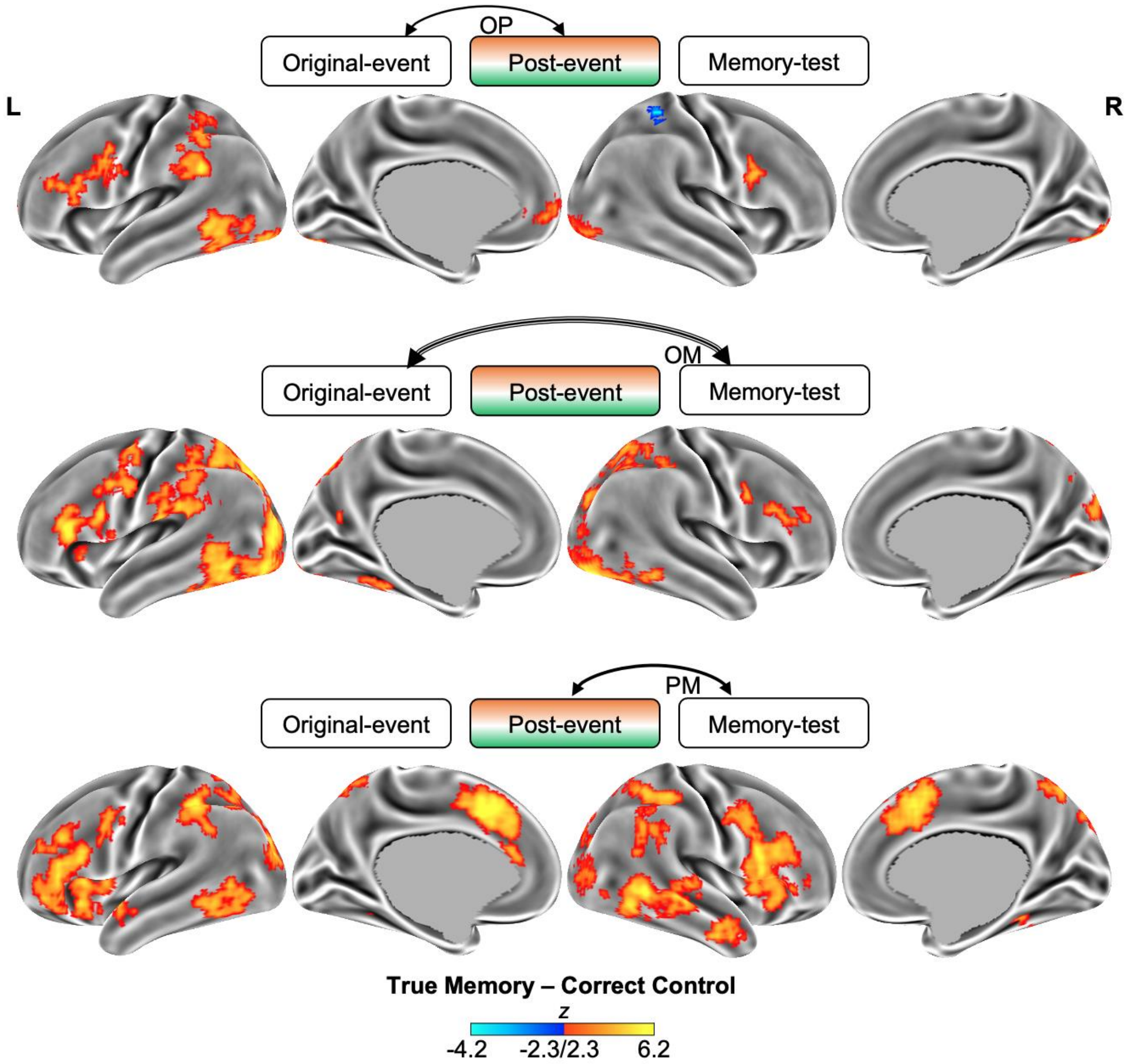


**Supplementary Figure 7. Brain regions showing differences between true memory and correct control in item-specific neural pattern similarity for OP (between original-event and post-event stages), OM (between original-event and memory-test stages), and PM (between post-event and memory-test stages) in Exp. 2 (thresholded at Z > 2.3).** Multiple brain regions (e.g., the left inferior frontal gyrus, left inferior parietal lobe, and left middle temporal gyrus) showed greater item-specific representations of information during the post-event and memory-test stages (i.e., OP, OM, and PM) for true memory than that for correct control. However, only the right superior parietal lobe showed greater item-specific representations of original information during the post-event stage (i.e., OP) for correct control than that for true memory.

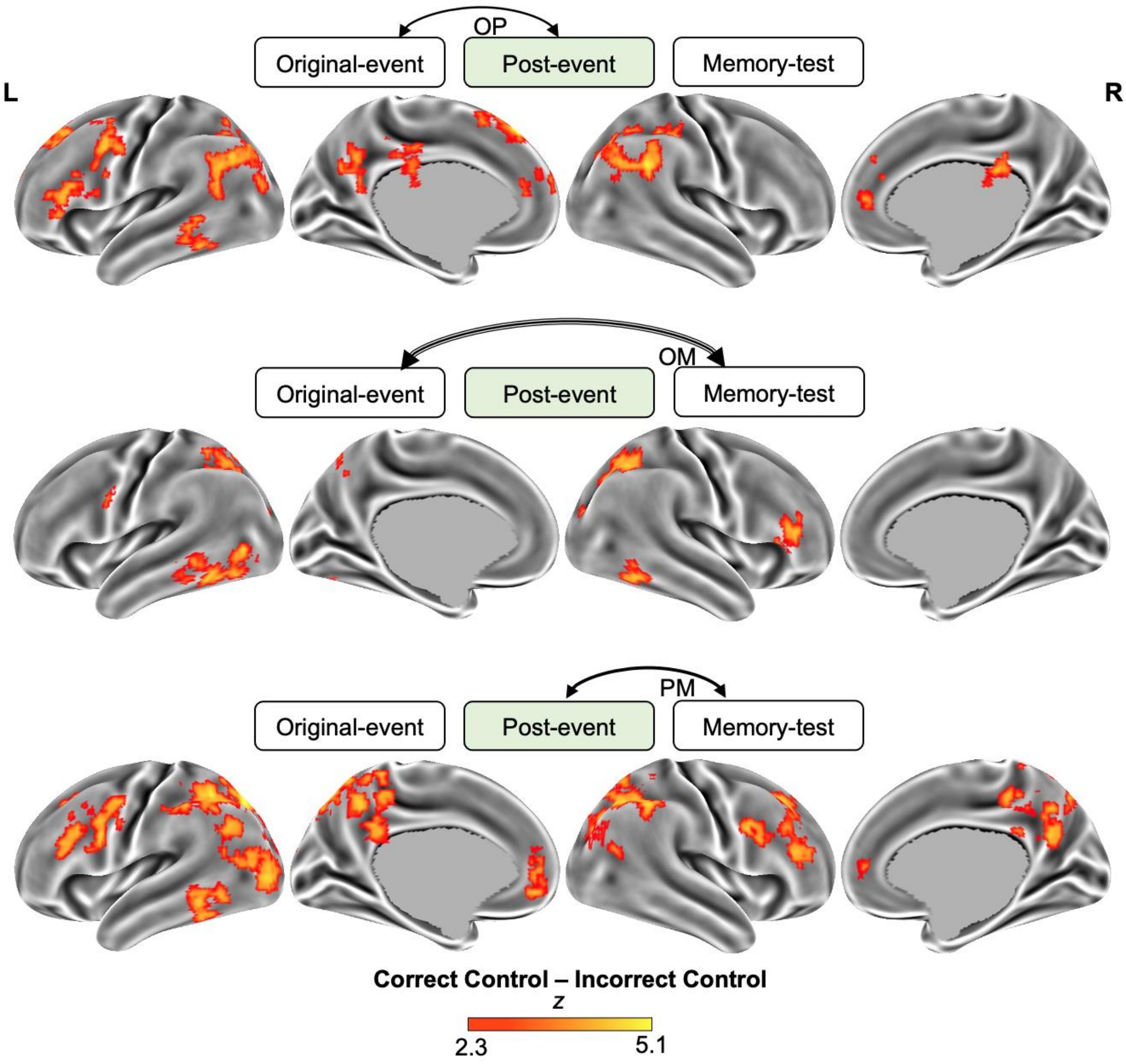


**Supplementary Figure 8. Brain regions showing differences between correct control and incorrect control in item-specific neural pattern similarity for OP (between original-event and post-event stages), OM (between original-event and memory-test stages), and PM (between post-event and memory-test stages), respectively in Exp. 2 (thresholded at Z > 2.3).** For each control item, its post-event narrative (e.g., reading "the lady was walking down the street, and there was a soda can next to her feet") was consistent with the corresponding original image (e.g., seeing "the lady was walking down the street, and there was a soda can next to her feet"). Thus, the contrast between correct and incorrect control reflects memory representations of consistent information. Several cortical regions (e.g., the bilateral superior parietal lobe and left middle temporal gyrus) showed greater item-specific representations for correct control compared to incorrect control. No region showed the reversed pattern.

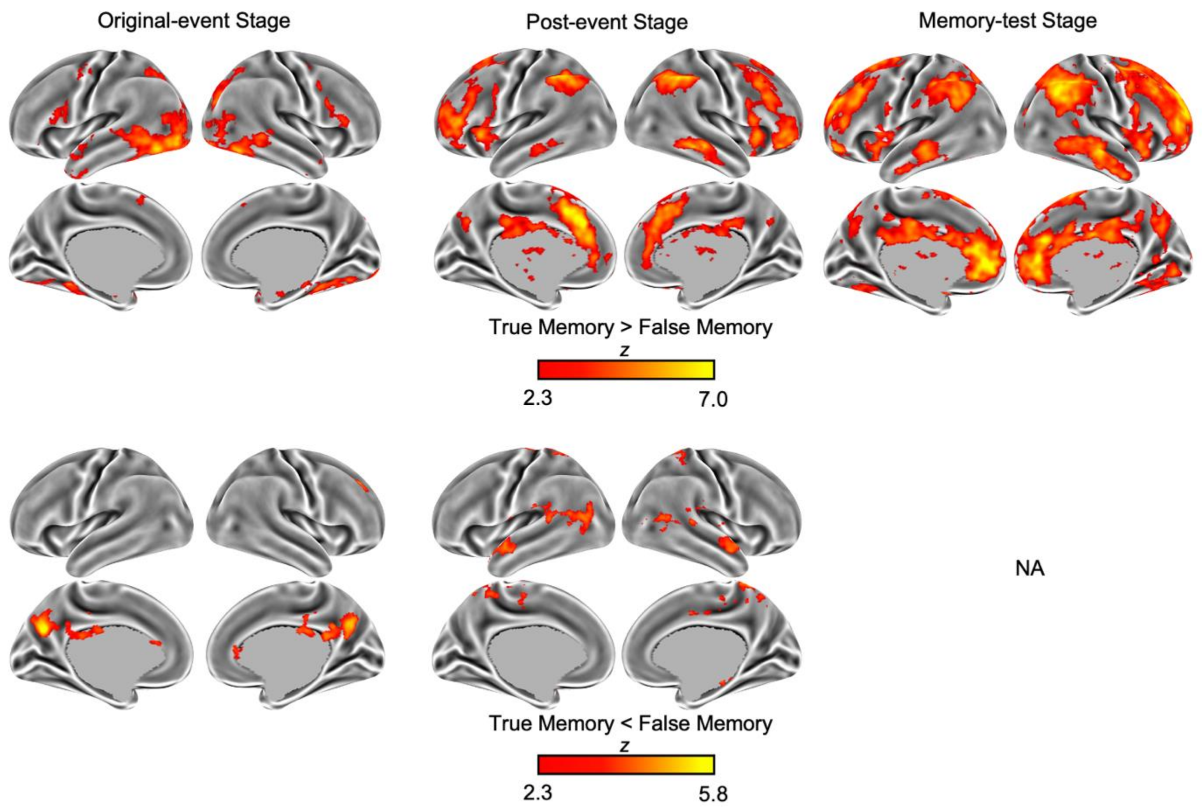


**Supplementary Figure 9. Brain regions showing univariate activation differences between true and false memories during the original-event, post-event, and memory-test stages in Exp. 2 (thresholded at Z > 2.3).**
Left: During the original-event stage, the bilateral frontal and visual cortex showed greater activations for subsequent true memory compared to false memory; whereas the precuneus and cingulate gyrus showed greater activations for subsequent false memory compared to true memory. These results suggested that, during the original-event stage, greater attention to visual details may support subsequent true memory than false memory[1], whereas greater attention to gist processing of the original events may support subsequent false memory than true memory.
Middle: During the post-event stage, the bilateral frontal, parietal, and middle temporal cortex, as well as medial frontal cortex and cingulate gyrus showed greater activations for subsequent true memory compared to false memory; whereas bilateral superior temporal cortex showed greater activations for subsequent false memory compared to true memory. These results suggested that, during the post-event stage, a greater prefrontal monitoring might support subsequent true memory than false memory[2,3], whereas a greater language processing might support false memory than true memory.
Right: During the memory-test stage, the bilateral frontal, parietal, and middle temporal cortex, as well as medial frontal cortex and cingulate gyrus showed greater activations of true memory compared to false memory; but no brain region showed greater activations of false memory compared to true memory. NA: not applicable. Those brain regions were located within the core recollection network, suggesting that true memory was associated with recovering more information than false memory during the memory-test stage[4].

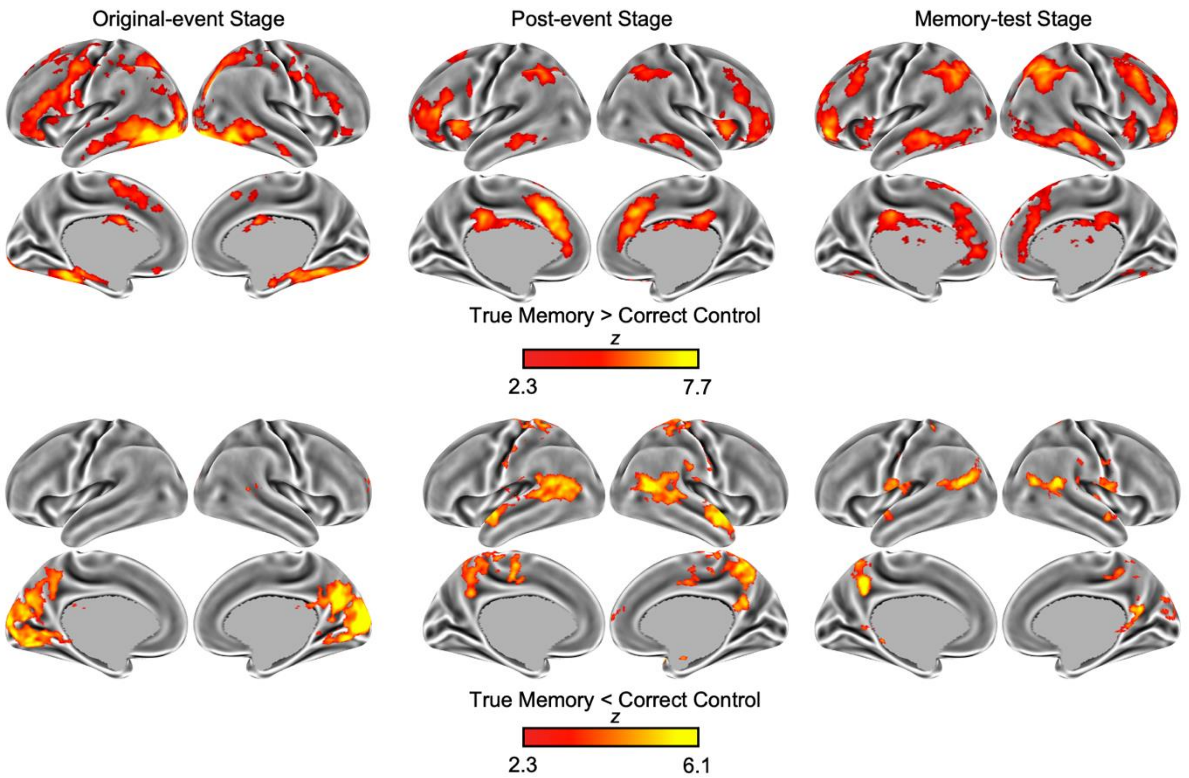


**Supplementary Figure 10. Brain regions showing univariate activation differences between true memory and correct control during the original-event, post-event, and memory-test stages in Exp. 2 (thresholded at Z > 2.3).** Left: During the original-event stage, the bilateral prefrontal cortex, parietal lobe, middle temporal gyrus, fusiform gyrus, and lateral occipital cortex showed greater activations for subsequent true memory compared to correct control; whereas the intracalcarine cortex, lingual gyrus, and occipital pole showed greater activations for subsequent correct control compared to true memory. These results suggested that, during the original-event stage, greater attention to high-level visual details (using the lateral visual cortex) might support subsequent true memory than correct control, whereas greater attention to low-level visual details (using the medial visual cortex) might support subsequent correct control than true memory.

Middle: During the post-event stage, the bilateral prefrontal cortex, paracingulate gyrus, inferior parietal lobe and middle temporal gyrus showed greater activations for subsequent true memory compared to correct control; whereas bilateral postcentral gyrus, superior temporal gyrus, and precuneus showed greater activations for subsequent correct control compared to true memory. Similar to the results from the contrast between true and false memories, the results from the contrast between true memory and correct control suggested that, during the post-event stage, a greater prefrontal monitoring might support subsequent true memory than correct control, whereas a greater language processing might support correct control than true memory.

Right: During the memory-test stage, the bilateral prefrontal cortex, inferior parietal lobe, middle temporal gyrus, and medial prefrontal cortex showed greater activations for true memory compared to correct control; whereas precuneus and bilateral superior temporal gyrus showed greater activations for correct control compared to true memory. It suggested that true memory was associated with recovering more visual information than correct control, while correct control was associated with recovering more semantic information than true memory during the memory-test stage.

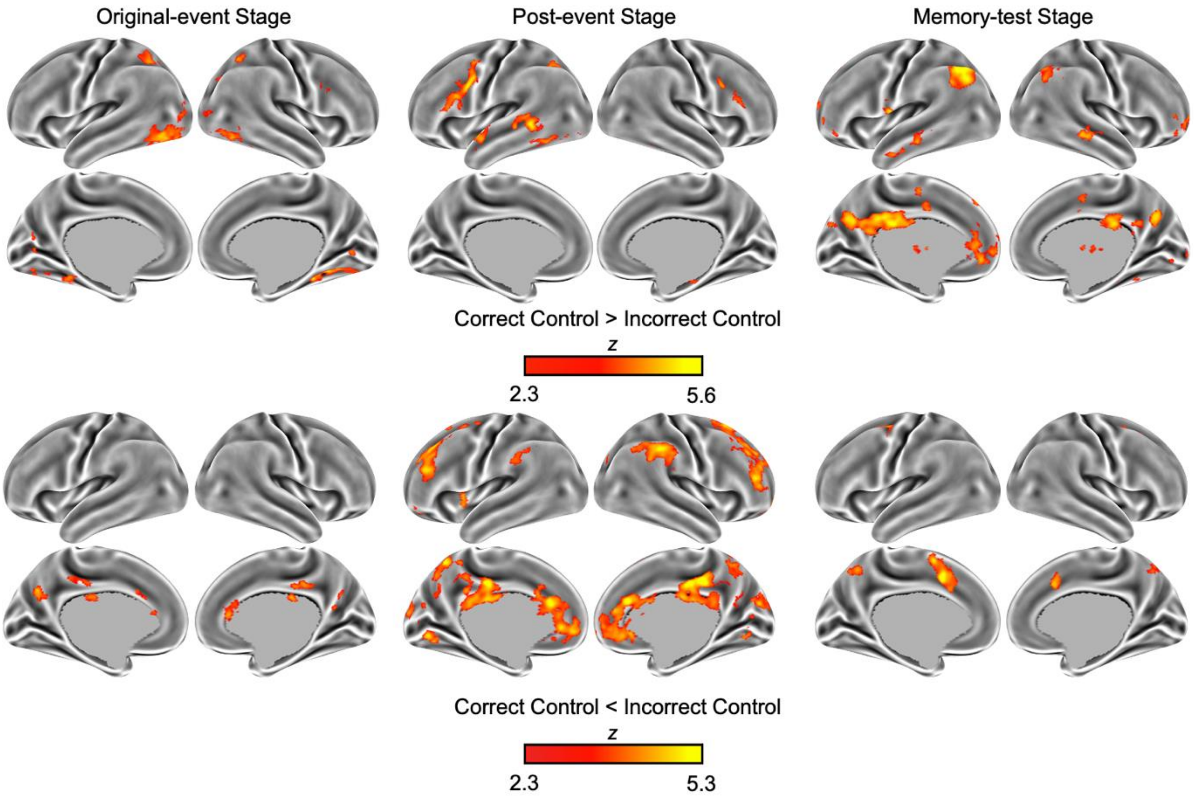


**Supplementary Figure 11. Brain regions showing univariate activation differences between correct control and incorrect control during the original-event, post-event, and memory-test stages (thresholded at Z > 2.3).**
Left: During the original-event stage, the bilateral visual cortex showed greater activations for subsequent correct control compared to incorrect control; whereas the bilateral cingulate gyrus showed greater activations for subsequent incorrect control compared to correct control. These results suggested that, during the original-event stage, greater attention to visual details might support subsequent correct control than incorrect control, whereas greater attention to gist processing might lead to subsequent incorrect control than correct control.
Middle: During the post-event stage, the bilateral middle frontal and left temporal gyrus showed greater activations for subsequent correct control compared to incorrect control; whereas the bilateral superior and medial frontal cortex, bilateral inferior parietal lobe, precuneus, and lingual gyrus showed greater activations for subsequent incorrect control compared to correct control. They might reflect the neural repetition enhancement and suppression during the post-event stage.
Right: During the memory-test stage, the medial prefrontal cortex, posterior cingulate gyrus, and bilateral inferior parietal lobe showed greater activations of correct control compared to incorrect control; whereas the bilateral superior frontal gyrus showed greater activations of incorrect control compared to correct control. It suggested that correct control was associated with recovering more information than incorrect control, while incorrect control was associated with more verification processes than correct control during the memory-test stage.

*Supplementary Tables*

Supplementary Table 1. Summary of prior and current fMRI studies exploring the neural basis of true memory (TM) and false memory (FM) during the original-event (OE), post-event (PE), and memory-test (MT) stages using the misinformation paradigm.

| Study & Participants | Analysis | Original-event | Post-event | Memory-test | Interval | Event order | Main findings |
|---|---|---|---|---|---|---|---|
| Okado et al. (2005)[2] *n* = 20 | Univariate **encoding** activation for subsequent memories | **(fMRI)** 8 criminal or daily events (50 images each) 3.5 seconds (sec) per image | **(fMRI)** 3.5 sec per image 96 critical items | Self-paced three-forced choice (3AFC) recognition test | OE-PE (10 min) PE-MT (2 days) | Random for OE. Same order for OE&MT | **Hippocampus** showed both item-encoding (i.e., OE: TM > FM and PE: FM > TM) and source-encoding pattern (i.e., PE: TM > FM and FM: OE > PE). |
| Baym et al. (2010)[1] *n* = 18 | Univariate **encoding** activation for subsequent memories | **(fMRI)** 12 daily events (50 images each) 2 sec per image | **(fMRI)** written narratives (self-paced imagery rating) 144 critical items | Self-paced 3AFC recognition test | OE-PE (1 hour) PE-MT (1 day) | Random for OE. Same order for OE&PE | Visual cortex (OE: TM>FM) Left insula (OE: FM>TM) Cingulate gyrus (PE: TM>FM) **Hippocampus** (OE/PE: TM=FM) |
| Jacques et al. (2013)[5] *n* = 26 | Univariate activation at **reactivation** for subsequent memories | A museum tour while wearing a camera 4 hours | **(fMRI)** 5 sec per photo making reliving rating and judging how related the two photos were | Y/N recognition test 56+16 lures (6 sec per question) | OE-PE (2 days) PE-MT (2 days) | Same order for OE, random order for PE | For target versus lure presentation, there was greater recruitment of posterior cingulate cortex and rostral medial PFC for hits, but greater recruitment of vmPFC and right **hippocampus** for false alarms |
| Edelson et al. (2011)[6] *n* = 20 | Univariate activation in memory **test** affected by **social conformity** | A movie watched in groups of 5 participants 40-min | **(fMRI for 2$^{nd}$ MT with PE)** Two-forced choice recognition test with four co-observer's misleading answers (self-paced) 80 critical items | | OE-1$^{st}$ MT (3 days) 1$^{st}$-2$^{nd}$ MT (4 days) 2$^{nd}$ -3$^{rd}$ MT (7 days) | PE and MT for each item were presented together | Greater activations for persistent errors than for transient errors and non-conformity in the amygdala and **hippocampus** during the 2$^{nd}$ memory test together with misinformation from co-observers. |
| Stark et al. (2010)[4] *n* = 25 | Univariate sensory reactivation in memory **test** | 11 criminal & daily events (50 images each) 3.5 sec per image | 1~3 sec per auditory narrative 132 critical items | **(fMRI)** Y/N recognition test (7 sec per question) | OE-PE (1 day) PE-MT (15 min) | Same order for three stages | Visual cortex (MT: TM>FM) Auditory cortex (MT: FM>TM) **Hippocampus** (MT: TM=FM) |
| Karanian et al. (2020)[7] *n* = 65 | Univariate sensory reactivation in the final **test** affected by **warning** | One silence video clip (criminal) 22-min | auditory narrative (135~160 words per min) 24 critical items | **(fMRI for 2$^{nd}$ MT)** 3AFC recognition test (7 sec per question) | OE-1$^{st}$ MT (immediate) 1$^{st}$ MT-PE (10 min) PE-2$^{nd}$ MT (immediate) | Same order for three stages | Visual cortex (MT: increased by warning to enhance TM) Auditory cortex (MT: decreased by warning to reduce TM) **Hippocampus** (MT: coordinated sensory cortical reactivations) |
| Current study (2023) *n* = 57 | **Multivariate**: Neural pattern similarity across three memory stages Item-specific | (**fMRI**) 8 criminal events (50 images each) 3.5 sec per image | (**fMRI**) 3.5 sec per written narrative 96 critical items 96 control items | (**fMRI**) 3AFC recognition test (9.5 sec per question) | OE-PE (3 hours) PE-MT (19 hours) | **Random** order across three stages | Cross-stage neural pattern similarity in the hippocampus predicted misinformation-induced false memory. |

*Note*. Due to the key role of the hippocampus in memory, prior studies have reported results of hippocampal univariate activations using fMRI data during one or two of three memory stages. However, their findings were mixed. It might be due to differences in their experimental design (e.g., stimulus duration, sensory modality, and interval).

Supplementary Table 2. Sex distribution and mean age for the three groups of participants in Exp. 1 (i.e., a behavioral experiment) and the misinformation group in Exp. 2 (i.e., a functional magnetic resonance imaging [fMRI] experiment using an independent sample).

| Group | *n* | Sex | | Age | |
|---|---|---|---|---|---|
| | | Female | Male | Mean ± SD | Range |
| Exp. 1 (behavioral) | | | | | |
| Misinformation | 40 | 21 | 19 | 21.43 ± 1.99 | (18 ~ 25) |
| Neutral | 42 | 22 | 20 | 20.76 ± 1.88 | (18 ~ 26) |
| Consistent | 40 | 20 | 20 | 20.60 ± 1.74 | (18 ~ 25) |
| Exp. 2 (fMRI) | | | | | |
| Misinformation | 57 | 29 | 28 | 21.94 ± 1.88 | (18 ~ 26) |

*Note.* Exp: experiment.

Among the three groups in Exp. 1, there was no sex difference ($\chi^2(2) = 0.064$, $p$ = 0.97, Cramer's V = 0.023) or age difference ($F(2, 119) = 2.19$, $p$ = 0.12, $\eta^2_p = 0.04$, 90% CI = [0.00, 0.10]). There was no sex difference ($\chi^2(2) = 0.0001$, $p$ = 0.99, Cramer's V = 0.0009) or age difference ($t(95) = 0.01$, $p$ = 0.99, $d$ = 0.002, 95% CI = [-0.40, 0.41]) in the two misinformation groups.

Supplementary Table 3. Options for critical and control items in the memory test for misinformation, neutral, and consistent groups.

| Group | Critical | | | Control | |
|---|---|---|---|---|---|
| | True (e.g., blue) | False (e.g., red) | Foil (e.g., white) | Correct (e.g., girl) | Incorrect (e.g., boy; old man) |
| Misinformation | OE only | Misinformation in PE | Not in OE or PE | OE & PE | Not in OE or PE |
| Neutral | OE only | Not in OE or PE | Not in OE or PE | OE only | Not in OE or PE |
| Consistent | OE & PE | Not in OE or PE | Not in OE or PE | OE & PE | Not in OE or PE |

*Note.* OE: details presented during the original-event stage. PE: details presented during the post-event stage. True: true memory of critical items. False: false memory of critical items. Foil: recognized foil of critical items. Correct: correct control items. Incorrect: incorrect control items.

Supplementary Table 4. The mean and standard deviation (Mean ± SD) for the endorsement rate in the memory test in two experiments.

| Group | Critical | | | Control | |
|---|---|---|---|---|---|
| | True | False | Foil | Correct | Incorrect |
| Exp. 1 (behavioral) | | | | | |
| Misinformation | 0.49 ± 0.08 | 0.41 ± 0.08 | 0.09 ± 0.04 | 0.68 ± 0.08 | 0.32 ± 0.07 |
| Neutral | 0.60 ± 0.06 | 0.25 ± 0.05 | 0.15 ± 0.04 | 0.56 ± 0.05 | 0.43 ± 0.05 |
| Consistent | 0.76 ± 0.08 | 0.15 ± 0.06 | 0.09 ± 0.04 | 0.69 ± 0.08 | 0.30 ± 0.08 |
| Exp. 2 (fMRI) | | | | | |
| Misinformation | 0.44 ± 0.07 | 0.42 ± 0.07 | 0.14 ± 0.04 | 0.60 ± 0.08 | 0.40 ± 0.08 |

*Note.* Exp: experiment. True: true memory of critical items. False: false memory of critical items. Foil: recognized foil of critical items. Correct: correct control items. Incorrect: incorrect control items.

Detailed results on endorsement rates:

In Exp. 1, the misinformation effect was demonstrated by greater differences between false memory and the endorsement rate of foils in the misinformation group than the neutral and consistent groups ($F(2, 119) = 141.07$, $p$ 4e$^{-32}$, $\eta^2_p = 0.70$, 90% CI = [0.63, 0.76]). Specifically, the misinformation group showed greater differences between false memory and foils compared to the neutral group ($t(80) = 12.39$ , $p$ 3e$^{-20}$, $d = 2.74$, 95% CI = [2.13, 3.34]), and the consistent group ($t(78) = 14.42$, $p$ 2e$^{-16}$, $d = 3.23$, 95% CI = [2.55, 3.89]). However, in Exp. 1, false memory was also higher than the endorsement rate of foils in the neutral group ($t(41) = 10.52$, $p$ 1e$^{-23}$, $d$ = 1.62, 95% CI = [1.16, 2.08]) and in the consistent group ($t(39) = 7.35$, $p$ = 7e$^{-9}$, $d$ = 1.16, 95% CI = [0.75, 1.56]), which may be caused by the design problem of the experimental material of foils. For critical items, the options of original information (e.g., a blue candy box) and misinformation (e.g., a red candy box) were counterbalanced across participants, but not for the foil option (e.g., a white candy box). Moreover, the reason that participants were less likely to select the foil option may be that they perceived the original and misinformation options were more reasonable than the foil option (e.g., red or blue candy boxes are more common than white candy boxes in real life). This did not affect the main results of the current study, because we examined the behavioral performance of misinformation effect by measuring the difference between false memory and the endorsement rate of foils rather than measuring false memory alone. Besides, true memory was higher than correct control in the neutral group ($t(41) = 4.43$, $p$ = 6e$^{-5}$, $d = 0.68$, 95% CI = [0.34, 1.02]) and in the consistent group ($t(39) = 8.06$, $p$ = 8e$^{-10}$, $d = 1.27$, 95% CI = [0.85,

1.69]). On the contrary, true memory was lower than correct control in the misinformation groups in Exp.1 ($t(39) = -14.57$, $p$ =2e$^{-17}$, $d = -2.30$, 95% CI = [-2.90, -1.70]) and in Exp. 2 ($t(56) = -12.27$, $p$ = 1e$^{-17}$, $d = -1.63$, 95% CI = [-2.02, -1.23]).

The misinformation groups in the two experiments did not differ in terms of the difference between false memory and the endorsement rate of foils ($F(1,95) = 3.52$, $p = 0.064$, $\eta^2_p = 0.04$, 90% CI = [0.00, 0.12]). There was no difference between the misinformation groups in two experiments in false memory ($t(95) = -0.73$, $p = 0.47$, $d = -0.15$, 95% CI = [-0.25, 0.56]), but the true memory and correct control were lower in Exp. 2 than those in Exp. 1 ($t(95) = -3.45$ and -4.94, $p$ = 8e$^{-4}$ and 3e$^{-6}$, $d = -0.71$ and -1.02, 95% CI = [-1.13, -0.29] and [-1.45, -0.59] for true memory and correct control, respectively), and the endorsement rates of foils and incorrect control were higher in Exp. 2 than those in Exp. 1 ($t(95) = 5.45$ and 5.04, $p$ = 4e$^{-7}$ and 2e$^{-6}$, $d$ = 1.12 and 1.04, 95% CI = [0.68, 1.55] and [0.61, 1.47] for foils and incorrect control, respectively). As reported in the main text, the misinformation effect was demonstrated in both experiments, and it was unaffected by the fMRI settings. However, true memory and correct control may be affected by the noisy MRI experimental environment, compared to the quiet behavioral experimental environment.

Supplementary Table 5. The mean and standard deviation (Mean ± SD) for the reaction time (RT) in the memory test in two experiments.

| Group | Critical | | Control | |
|---|---|---|---|---|
| | True | False | Correct | Incorrect |
| Exp. 1 (behavioral) | | | | |
| Misinformation | 0.79 ± 0.20 | 0.85 ± 0.24 | 0.82 ± 0.25 | 1.00 ± 0.30 |
| Neutral | 0.79 ± 0.16 | 0.95 ± 0.22 | 0.84 ± 0.18 | 0.98 ± 0.21 |
| Consistent | 0.70 ± 0.18 | 0.97 ± 0.29 | 0.77 ± 0.17 | 0.98 ± 0.26 |
| Exp. 2 (fMRI) | | | | |
| Misinformation | 0.77 ± 0.20 | 0.78 ± 0.20 | 0.76 ± 0.19 | 0.89 ± 0.24 |

*Note.* Exp: experiment. True: true memory of critical items. False: false memory of critical items. Correct: correct control items. Incorrect: incorrect control items.

Detailed results on reaction time:

For the reaction time (RT) during the memory test in Exp. 1, there was a significant interaction between group type (i.e., misinformation, neutral, and consistent) and memory type (i.e., true, false, correct, and incorrect) ($F$(6, 357) = 9.09, $p$ =4e$^{-8}$, $\eta^2_p$ = 0.13, 90% CI = [0.07, 0.18]). The mean RT of false memory in the misinformation group was shorter than that in the consistent group ($t$(78) = -2.02, $p$ = 0.046, $d$ = -0.45, 95% CI = [-0.89, -0.01]), the mean RT of true memory in the consistent group was shorter than those in the neutral group ($t$(80) = -2.17, $p$ = 0.032, $d$ = -0.48, 95% CI = [-0.92, -0.04]) and the misinformation group ($t$(78) = -2.03, $p$ = 0.045, $d$ = -0.44, 95% CI = [-0.88, -0.01]). No group difference was found for the RT of correct control or incorrect control ($ps$ > 0.13). In the memory test for the misinformation group of Exp. 1, the mean RT of incorrect control was longer than that of correct control, true memory, and false memory ($t$(39) = 8.41, 8.29, and 6.27, $p$ = 3e$^{-10}$ ,4e$^{-10}$, 2e$^{-7}$ , $d$ = 1.33, 1.31, and 0.99, 95% CI = [0.90, 1.75], [0.88, 1.73], and [0.61, 1.37] for correct control, true memory, and false memory, respectively), and the mean RT of false memory was longer than that of true memory ($t$(39) = 3.03, $p$ = 0.004, $d$ = 0.48, 95% CI = [0.15, 0.80]). In the memory test of Exp. 2, the mean RT of incorrect control was longer than that of correct control, true memory, and false memory ($t$(56) = 6.43, 5.84, and 5.94, $p$ = 3e$^{-8}$, 3e$^{-7}$, 2e$^{-7}$, $d$ = 0.85, 0.77, and 0.79, 95% CI = [0.55, 1.15], [0.47, 1.07], and [0.49, 1.08] for correct control, true memory, and false memory, respectively). There was no group difference in these indices between the

misinformation groups in the two experiments (*ps* > 0.16), except that the mean RT of incorrect control in Exp. 1 was longer than that in Exp. 2 ($t(95) = 2.10$, $p = 0.047$, $d = 0.43$, 95% CI = [0.02, 0.84]).

Supplementary Table 6. The mean and standard deviation (Mean ± SD) for the hippocampal pattern similarity across the three stages for corresponding items, non-corresponding items in the same event, and item-specific representation for each memory type, and for the hippocampal univariate activation level (percent signal change) during the three stages in Exp. 2.

| | Critical | | Control | |
|---|---|---|---|---|
| | True | False | Correct | Incorrect |
| Neural pattern similarity | | | | |
| OP | | | | |
| corresponding | 0.0055 ± 0.0087 | 0.0037 ± 0.0110 | 0.0036 ± 0.0081 | 0.0042 ± 0.0096 |
| non-corresponding | 0.0009 ± 0.0051 | 0.0017 ± 0.0040 | 0.0015 ± 0.0041 | 0.0016 ± 0.0041 |
| item-specific | **0.0046 ± 0.0081** | **0.0020 ± 0.0097** | **0.0021 ± 0.0071** | **0.0026 ± 0.0091** |
| OM | | | | |
| corresponding | 0.0016 ± 0.0090 | 0.0037 ± 0.0087 | 0.0043 ± 0.0082 | 0.0020 ± 0.0095 |
| non-corresponding | 0.0012 ± 0.0060 | 0.0017 ± 0.0059 | 0.0014 ± 0.0059 | 0.0020 ± 0.0056 |
| item-specific | **0.0004 ± 0.0070** | **0.0020 ± 0.0073** | **0.0029 ± 0.0071** | **0.00004 ± 0.0080** |
| PM | | | | |
| corresponding | 0.0014 ± 0.0097 | 0.0058 ± 0.0096 | 0.0047 ± 0.0096 | 0.0032 ± 0.0105 |
| non-corresponding | 0.0016 ± 0.0050 | 0.0022 ± 0.0050 | 0.0016 ± 0.0050 | 0.0021 ± 0.0053 |
| item-specific | **-0.0002 ± 0.0079** | **0.0036 ± 0.0076** | **0.0031 ± 0.0082** | **0.0011 ± 0.0099** |
| Univariate activation level | | | | |
| Original-event stage | 0.0300 ± 0.0880 | 0.0289 ± 0.0931 | 0.0193 ± 0.0774 | 0.0239 ± 0.0950 |
| Post-event stage | -0.0429 ± 0.0992 | -0.0212 ± 0.1072 | -0.0322 ± 0.0929 | -0.0340 ± 0.1104 |
| Memory-test stage | -0.1267 ± 0.1141 | -0.1310 ± 0.1244 | -0.1238 ± 0.1221 | -0.1205 ± 0.1283 |

*Note.* OP: Neural pattern similarity between original-event and post-event stages. OM: Neural pattern similarity between original-event and memory-test stages. PM: Neural pattern similarity between post-event and memory-test stages. Bolded scores represent item-specific neural pattern similarity, which was calculated by the neural pattern similarity for corresponding items minus that for non-corresponding items in the same event for each type of memory. True: true memory of critical items. False: false memory of critical items. Correct: correct control items. Incorrect: incorrect control items.

(1) Additional results of hippocampal pattern similarity:

To directly compare false memory and correct control, we conducted a 2 (memory types: false memory vs. correct control) × 3 (stage pairs) × 2 (item specificity) repeated measures ANOVA. Results showed that only the main effect of item specificity was significant ($F$(1, 56) = 26.79, $p$ = 3e$^{-6}$, $\eta^2_p$ = 0.32, 90% CI = [0.16, 0.47]), while none of the other main effects or interaction terms was significant ($ps$ > 0.39). Thus, we combined false memory and correct control, and compared them with true memory using the 2 (memory types: [false memory & correct control] vs. true memory) × 3 (stage pairs) × 2 (item specificity) repeated measures ANOVA. Results showed a significant three-way interaction ($F$(2, 112) = 6.31, $p$ = 0.003, $\eta^2_p$ = 0.10, 90% CI = [0.02, 0.19]), a significant two-way interaction between stage pair and item specificity ($F$(2, 112) = 3.39, $p$ = 0.037, $\eta^2_p$ = 0.06, 90% CI = [0.001, 0.13]), and significant main effects of item specificity and memory type ($F$(1, 56) = 25.94 and 6.15, $p$ = 4e$^{-6}$ and 0.016, $\eta^2_p$ = 0.32 and 0.10, 90% CI = [0.16, 0.46] and [0.01, 0.24]), while the main effect of stage pair and the other two-way interactions were not significant in this model ($ps$ > 0.06). To probe this three-way interaction, we conducted two-way ANOVA by stage pair. Only for PM, there were a significant two-way interaction between memory type and item specificity ($F$(1, 56) = 7.79, $p$ = 0.007, $\eta^2_p$ = 0.12, 90% CI = [0.02, 0.26), and significant main effects of memory type and item specificity ($F$(1, 56) = 6.76 and 5.10, $p$ = 0.012 and 0.028, $\eta^2_p$ = 0.11 and 0.08, 90% CI = [0.01, 0.25] and [0.01, 0.22]). Simple effect analysis for PM showed that hippocampal pattern similarity was higher for corresponding items than for non-corresponding items in the combined memory type of false memory and correct control ($t$(56) = 4.29, $p$ = 7e$^{-5}$, $d$ = 0.57, 95% CI = [0.29, 0.85]), but not in true memory ($p$ = 0.82). For OP, the main effect of item specificity was significant ($F$(1, 56) = 23.82, $p$ = 9e$^{-6}$, $\eta^2_p$ = 0.30, 90% CI = [0.14, 0.44]), but the main effect of memory type and the interaction were not significant ($ps$ > 0.05). For OM, there were significant main effects of item specificity ($F$(1, 56) = 4.48, $p$ = 0.039, $\eta^2_p$ = 0.07, 90% CI = [0.001, 0.20] [corresponding > non-corresponding]) and memory type ($F$(1, 56) = 7.41, $p$ = 0.009, $\eta^2_p$ = 0.12, 90% CI = [0.02, 0.26] [false memory & correct control > true memory]), but their interaction was not significant ($p$ = 0.089).

In addition, we examined the memory effect for control items by comparing correct control and incorrect control on item-specific hippocampal pattern similarity in OM or in PM. For OM, the effect of item specificity for correct control was higher than for incorrect control ($t$(56) = 2.02, $p$ = 0.048, $d$ = 0.27, 95% CI = [0.002, 0.53]). For PM, however, the effect of item specificity for correct control was not significantly higher than for incorrect control ($t$(56) = 1.21, $p$ = 0.23, $d$ = 0.16, 95% CI = [-0.10, 0.42]).

(2) Additional results using partial correlations for PM and OM:

Partial correlations were calculated for PM and OM at the trial level for corresponding and non-corresponding items, separately. For the corresponding items, a partial correlation for PM was calculated between the neural pattern of one item during the memory-test stage (e.g., test item 26) and the neural pattern of its corresponding narrative during the post-event stage (e.g., narrative 26), after controlling for the neural pattern of its corresponding image during the original-test stage (e.g., image 26). For the non-corresponding items in the same event, partial correlations for PM were calculated between neural patterns during the memory-test (e.g., test item 26) and post-event stages (e.g., narrative 1), after controlling for that during the original-test stage (e.g., image 1). Then, these similarity scores were transformed into Fisher's Z scores, which were then averaged to generate the neural pattern similarity value for each type of trial. The same method was used to calculate partial correlations for OM, except that neural pattern similarities were computed between original-event and memory-test stages after controlling for the neural pattern during the post-event stage. It should be noted that we did not calculate partial correlations for OP, because the memory test was conducted after the original and post-event stages (i.e., OP was unlikely to be influenced by neural patterns during the subsequent memory test). Using the partial correlations for PM and OM and original correlations for OP, we conducted the 3 by 4 by 2 repeated measures ANOVA on the hippocampal pattern similarity.

These additional analyses confirmed the significant results as reported in Fig. 3d in the main text. A 3 (stage pairs) × 4 (memory types) × 2 (item specificity) repeated measures ANOVA showed a significant three-way interaction ($F(6, 336) = 2.15$, $p = 0.047$, $\eta^2_p = 0.04$, 90% CI = [0.001, 0.06]), and a significant main effect of item specificity ($F(1, 56) = 25.70$, $p = 5e^{-6}$, $\eta^2_p = 0.31$, 90% CI = [0.16, 0.46]), while the other main effects and the two-way interactions were not significant (*ps* > 0.13). We then analyzed the data by memory type. For true memory, the two-way interaction between stage pair and item specificity was significant ($F(2, 112) = 5.86$, $p = 0.004$, $\eta^2_p = 0.09$, 90% CI = [0.02, 0.18]). Simple effect analysis for true memory showed that the hippocampal pattern similarity was higher for corresponding items than for non-corresponding items in OP ($t(56) = 4.33$, $p = 6e^{-5}$, $d = 0.57$, 95% CI = [0.29, 0.85]) but not in OM or PM (*ps* > 0.77), and the effect of item specificity was greater in OP than in OM and PM ($t(56)$ = 3.22 and 2.84, $p$ = 0.002 and 0.006, $d$ = 0.43 and 0.38, 95% CI = [0.15, 0.70] and [0.11, 0.64]). For false memory, the effect of item specificity was significant ($F(1, 56) = 17.61$, $p = 0.0001$, $\eta^2_p = 0.24$, 90% CI = [0.09, 0.39]), but the effect of stage pair and the interaction between stage pair and item specificity were not significant (*ps* > 0.46). For correct control, the effect of item specificity was significant ($F(1, 56) = 14.60$, $p = 0.0003$, $\eta^2_p = 0.21$, 90% CI = [0.07,

0.36]), but the effect of stage pair and the interaction between stage pair and item specificity were not significant (*ps* > 0.76). Finally, for incorrect control, none of the effects (stage pair, item specificity, or their interaction) was significant (*ps* > 0.06). For PM, hippocampal pattern similarity was higher for corresponding items than that for non-corresponding items for false memory ($t(56) = 3.48$, $p = 0.0009$, $d = 0.46$, 95% CI = [0.19, 0.73]) but not for true memory ($p = 0.97$), and the effect of item specificity in PM was greater for false memory than for true memory ($t(56) = 2.60$, $p = 0.012$, $d = 0.34$, 95% CI = [0.08, 0.61]). These additional results still support the multiple-trace theory (i.e., two distinct memory traces during the memory test).

(3) Results of hippocampal univariate activation level:

Using a 3 (stage type) × 4 (memory type) repeated measures ANOVA to examine the effect of stage type and memory type on the hippocampal activation level, only the main effect of stage type was significant ($F(2, 112) = 39.67$, $p = 2e^{-13}$, $\eta^2_p = 0.41$, 90% CI = [0.30, 0.51]), while the main effect of memory type and the interaction were not significant (*ps* > 0.24). Generally, the hippocampal activation level during the original-event stage was higher than those during the post-event stage ($t(56) = 3.99$, $p = 0.0002$, $d = 0.53$, 95% CI = [0.25, 0.80]), which in turn was higher than those during the memory test ($t(56) = 5.10$, $p = 4e^{-6}$, $d = 0.68$, 95% CI = [0.39, 0.96]).

Supplementary Table 7. P values for the comparisons of correlations for hippocampal item-specific representations (before and after corrections) in Exp. 2.

| | Critical | | Control | |
|---|---|---|---|---|
| | True | False | Correct | Incorrect |
| OP vs. OM | | | | |
| Before correction | **0.003** | 0.997 | 0.542 | 0.120 |
| After correction | **0.003** | 0.979 | 0.547 | 0.120 |
| OM vs. PM | | | | |
| Before correction | 0.645 | 0.238 | 0.907 | 0.532 |
| After correction | 0.717 | 0.247 | 0.909 | 0.474 |
| OP vs. PM | | | | |
| Before correction | **0.002** | 0.314 | 0.498 | 0.360 |
| After correction | **0.002** | 0.338 | 0.499 | 0.389 |

*Note*. OP: Neural pattern similarity between original-event and post-event stages.
OM: Neural pattern similarity between original-event and memory-test stages.
PM: Neural pattern similarity between post-event and memory-test stages.
Item-specific neural pattern similarity was calculated by the neural pattern similarity for the corresponding items minus that for the non-corresponding items in the same event for each memory type. Bolded scores represent significant *p* values. True: true memory of critical items. False: false memory of critical items. Correct: correct control items. Incorrect: incorrect control items.

Using the method of Meng, et al.[8], we reported *p* values after correction for comparing correlated correlation coefficients. We used the cocor package (version 1.1-4), which is a software package for the R programming language[9]. The results after correction were virtually the same as those reported in the main text (i.e., before correction).

Methods in detail:

(1) OP vs. OM for item-specific representation

Meng's z scores for OP vs. OM for the corresponding items:
For each item in each participant, we calculated Meng's z score by comparing its OP and OM while controlling for its PM for corresponding items. The sample size for these three correlations was the number of voxels in the bilateral hippocampus for each participant (voxel size of bilateral hippocampus: 1144 ± 109, max = 1373, min = 842). Next, Meng's z scores for OP vs. OM for corresponding items were averaged for all items for each participant.

Meng's z scores for OP vs. OM for the non-corresponding items in the same event: For each item in each participant, we calculated Meng's z score by comparing its OP and OM with each one of 49 non-corresponding items in the same event while controlling for its PM. Next, Meng's z scores for OP vs. OM for non-corresponding items in the same event were averaged for all items for each participant.

Finally, in order to test the contrast of OP vs. OM (for item-specific representation), the paired *t*-test was used to compare Meng' s z scores for OP vs. OM (for corresponding items) and for OP vs. OM (for non-corresponding items in the same event).

(2) OM vs. PM for item-specific representation

The same method was used as described above, except that comparisons between OM and PM were controlled for OP.

(3) OP vs. PM for item-specific representation

The same method was used as described above, except that comparisons between OP and PM were controlled for OM.

Supplementary Table 8. The mean and standard deviation (Mean ± SD) for the anterior and posterior hippocampal pattern similarity across the three stages for corresponding items, non-corresponding items in the same event, and item-specific representation for each memory type in Exp. 2.

| | Critical | | Control | |
|---|---|---|---|---|
| | True | False | Correct | Incorrect |
| Anterior hippocampus | | | | |
| OP | | | | |
| corresponding | 0.0061 ± 0.0792 | 0.0042 ± 0.0784 | 0.0048 ± 0.0788 | 0.0033 ± 0.0805 |
| non-corresponding | 0.0014 ± 0.0305 | 0.0021 ± 0.0302 | 0.0018 ± 0.0312 | 0.0014 ± 0.0303 |
| item-specific | **0.0047 ± 0.0759** | **0.0021 ± 0.0745** | **0.0030 ± 0.0745** | **0.0019 ± 0.0750** |
| OM | | | | |
| corresponding | 0.0005 ± 0.0799 | 0.0010 ± 0.0797 | 0.0024 ± 0.0793 | 0.0034 ± 0.0811 |
| non-corresponding | 0.0008 ± 0.0310 | 0.0013 ± 0.0312 | 0.0006 ± 0.0313 | 0.0016 ± 0.0306 |
| item-specific | **-0.0003 ± 0.0751** | **-0.0003 ± 0.0745** | **0.0018 ± 0.0749** | **0.0018 ± 0.0768** |
| PM | | | | |
| corresponding | 0.0035 ± 0.0789 | 0.0053 ± 0.0803 | 0.0041 ± 0.0798 | -0.0006 ± 0.0808 |
| non-corresponding | 0.0017 ± 0.0296 | 0.0019 ± 0.0304 | 0.0019 ± 0.0306 | 0.0016 ± 0.0308 |
| item-specific | **0.0018 ± 0.0743** | **0.0034 ± 0.0740** | **0.0022 ± 0.0754** | **-0.0022 ± 0.0774** |
| Posterior hippocampus | | | | |
| OP | | | | |
| corresponding | 0.0002 ± 0.0822 | 0.0008 ± 0.0806 | -0.0014 ± 0.0807 | 0.0004 ± 0.0798 |
| non-corresponding | -0.0009 ± 0.0311 | -0.0004 ± 0.0310 | -0.00003 ± 0.0313 | -0.0004 ± 0.0304 |
| item-specific | **0.0011 ± 0.0778** | **0.0012 ± 0.0759** | **-0.0014 ± 0.0771** | **0.0008 ± 0.0762** |
| OM | | | | |
| corresponding | 0.0002 ± 0.0836 | 0.0036 ± 0.0823 | 0.0041 ± 0.0824 | -0.0003 ± 0.0818 |
| non-corresponding | 0.0014 ± 0.0317 | 0.0015 ± 0.0309 | 0.0009 ± 0.0309 | 0.0016 ± 0.0310 |
| item-specific | **-0.0012 ± 0.0792** | **0.0021 ± 0.0774** | **0.0032 ± 0.0777** | **-0.0019 ± 0.0768** |
| PM | | | | |
| corresponding | -0.0030 ± 0.0835 | 0.0008 ± 0.0817 | 0.0031 ± 0.0823 | 0.0022 ± 0.0818 |
| non-corresponding | -0.0010 ± 0.0308 | -0.0005 ± 0.0310 | -0.0012 ± 0.0307 | 0.0005 ± 0.0306 |
| item-specific | **-0.0020 ± 0.0786** | **0.0013 ± 0.0767** | **0.0043 ± 0.0783** | **0.0017 ± 0.0779** |

*Note.* OP: Neural pattern similarity between original-event and post-event stages. OM: Neural pattern similarity between original-event and memory-test stages. PM: Neural pattern similarity between post-event and memory-test stages. Bolded scores represent item-specific neural pattern similarity, which was calculated by the neural pattern similarity for corresponding items minus that for non-corresponding items in the same event for each type of memory. True: true memory of critical items. False: false memory of critical items. Correct: correct control items. Incorrect: incorrect control items. For each participant, the whole hippocampus was divided into its anterior and posterior segments based on the location of uncal apex in the native space.

Results of anterior and posterior hippocampal pattern similarity:

The 3 (stage pairs) × 4 (memory types) × 2 (item specificity) × 2 (ROI: anterior and posterior hippocampus) repeated-measured ANOVA showed a significant four-way interaction ($F$(6, 336) = 2.19, $p$ = 0.044, $\eta^2_p$ = 0.04, 90% CI = [0.001, 0.06]), and significant main effects of item specificity and ROI ($F$(1, 56) = 9.75 and 6.07, $p$ = 0.003 and 0.017 , $\eta^2_p$ = 0.15 and 0.10, 90% CI = [0.03, 0.29] and [0.01, 0.23]), while the other main effects or interaction terms were not significant (*ps* > 0.059). To interpret the four-way interaction, we examined the results by memory type. Only for correct control, there was a significant three-way interaction among stage pair, item specificity, and ROI ($F$(2, 112) = 4.21, $p$ = 0.017, $\eta^2_p$ = 0.07, 90% CI = [0.01, 0.15]). However, this three-way interaction was not significant for true memory, false memory, or incorrect control (*ps* > 0.064). To further interpret this three-way interaction for correct control, we examined the data by stage pair. There was a significant two-way interaction between item specificity and ROI in OP ($F$(1, 56) = 4.43, $p$ = 0.040, $\eta^2_p$ = 0.07, 90% CI = [0.001, 0.20]), but not in OM and PM (*ps* > 0.25). Simple effect analysis showed that the neural pattern similarity for correct control in OP was marginally higher for corresponding items than non-corresponding items in the anterior hippocampus ($t$(56) = 1.98, $p$ = 0.052, $d$ = 0.26, 95% CI = [0.001, 0.53]), but not in the posterior hippocampus ($t$(56) = -0.96, $p$ = 0.34, $d$ = -0.13, 95% CI = [-0.39, 0.13]). For true memory, although the three-way interaction among stage pair, item specificity, and ROI was not significant, there was a significant two-way interaction between item specificity and ROI ($F$ (1, 56) = 7.24, $p$ = 0.009, $\eta^2_p$ = 0.11, 90% CI = [0.02, 0.25]). Simple effect analysis showed that neural pattern similarity was higher for corresponding items than non-corresponding items in the anterior hippocampus ($t$(56) = 2.52, $p$ = 0.014, $d$ = 0.33, 95% CI = [0.07, 0.60]), but not in the posterior hippocampus ($t$(56) = -0.87, $p$ = 0.39, $d$ = -0.11, 95% CI = [-0.37, 0.15]). For false memory and incorrect control, none of these effects or their interaction was significant, except for the main effect of item specificity for false memory ($F$ (1, 56) = 5.60, $p$ = 0.021, $\eta^2_p$ = 0.09, 95% CI = [0.01, 0.23]). These

results indicated a functional dissociation along the long axis of the hippocampus for true memory and correct control rather than for false memory and incorrect control.

Supplementary Table 9. The means and standard deviations (Mean ± SD) for the Fisher's Z scores of within-participant and between-participant correlations between hippocampal item-specific representation and behavioral performance of false memory, correct control, or true memory in Exp. 2.

| | Correlations with PM | | Correlations with OM | |
|---|---|---|---|---|
| | Within -participant | Between -participant | Within -participant | Between -participant |
| False memory | **0.022 ± 0.067** | **-0.005 ± 0.040** | 0.006 ± 0.063 | -0.002 ± 0.038 |
| Correct control | 0.011 ± 0.084 | 0.005 ± 0.056 | 0.013 ± 0.073 | 0.004 ± 0.050 |
| True memory | *-0.019 ± 0.066* | *-0.002 ± 0.046* | -0.009 ± 0.067 | 0.003 ± 0.039 |

*Note.* OM: Neural pattern similarity between original-event and memory-test stages.

PM: Neural pattern similarity between post-event and memory-test stages.

Item-specific neural pattern similarity was calculated by the neural pattern similarity for the corresponding items minus that for the non-corresponding items in the same event for each memory type. Greater within-participant than between-participant neural-behavioral correlations are shown in bold. Lower within-participant than between-participant neural-behavioral correlations are shown in italics. As shown by a previous study[10], the results of individuation analysis are meaningful only when the within-participant correlation is greater than the between-participant correlation. The negative within-participant correlation for true memory contradicts the premise of the individuation analysis. Therefore, we are not concerned with this negative correlation.

Supplementary Table 10. Brain regions showing participant-specific neural-behavioral correlations for true memory, false memory, and correct control in Exp. 2 (thresholded at Z > 2.3).

| Neural-behavioral correlation | Region | Z | x | y | z |
|---|---|---|---|---|---|
| Correlations with OM | | | | | |
| True memory | | | | | |
| | Left superior parietal lobe | 4.3 | -38 | -74 | 46 |
| | Right superior parietal lobe | 4.5 | 38 | -62 | 54 |
| | Left angular gyrus | 4.3 | -46 | -68 | 28 |
| | Left middle temporal gyrus | 3.5 | -58 | -54 | -8 |
| False memory | | | | | |
| | NA | | | | |
| Correct control | | | | | |
| | NA | | | | |
| Correlations with PM | | | | | |
| True memory | | | | | |
| | NA | | | | |
| False memory | | | | | |
| | Right frontal pole | 3.6 | 34 | 54 | 28 |
| | Right middle frontal gyrus | 3.8 | 36 | 26 | 50 |
| | Left superior frontal gyrus | 3.7 | -18 | 36 | 40 |
| | Anterior cingulate cortex | 4.4 | 6 | 34 | 26 |
| | Right supramarginal gyrus | 4.8 | 54 | -44 | 54 |
| | Posterior cingulate cortex | 4.1 | 4 | -16 | 46 |
| | Right hippocampus | 2.8 | 36 | -26 | -14 |
| Correct control | | | | | |
| | NA | | | | |

*Note.* Not applicable. OM: Neural pattern similarity between original-event and memory-test stages. PM: Neural pattern similarity between post-event and memory-test stages.

Supplementary Table 11. Brain regions showing differences in item-specific neural pattern similarity between true and false memories for OP (between original-event and post-event stages), OM (between original-event and memory-test stages), and PM (between post-event and memory-test stages) in Exp. 2 (thresholded at Z > 2.3).

| Contrast | Region | Z | x | y | z |
|---|---|---|---|---|---|
| Neural pattern similarity between original-event and post-event stages (OP: item-specific representation) | | | | | |
| True > False | | | | | |
| | Left inferior frontal gyrus | 4.3 | -46 | 32 | 18 |
| | Right frontal pole | 3.5 | 42 | 54 | 2 |
| | Medial prefrontal cortex | 3.8 | 6 | 46 | 20 |
| | Left inferior parietal lobe | 3.9 | -62 | -38 | 32 |
| | Left middle temporal gyrus | 4.6 | -58 | -50 | -8 |
| False > True | | | | | |
| | NA | | | | |
| Neural pattern similarity between original-event and memory-test stages (OM: item-specific representation) | | | | | |
| True > False | | | | | |
| | Left inferior frontal gyrus | 3.9 | -50 | 36 | 10 |
| | Left superior frontal gyrus | 3.6 | -20 | 30 | 60 |
| | Medial prefrontal cortex | 3.5 | -10 | 54 | 4 |
| | Left precentral gyrus | 4.4 | -48 | 2 | 26 |
| | Right superior parietal lobe | 5.3 | 38 | -62 | 54 |
| | Left inferior parietal lobe | 5.4 | -48 | -46 | 42 |
| | Posterior cingulate gyrus | 3.7 | -6 | -28 | 40 |
| | Left inferior temporal gyrus | 4.7 | -50 | -54 | -10 |
| | Right inferior temporal gyrus | 4.4 | 58 | -54 | -18 |
| False > True | | | | | |
| | NA | | | | |
| Neural pattern similarity between post-event and memory-test stages (PM: item-specific representation) | | | | | |
| True > False | | | | | |
| | Right inferior frontal gyrus | 4.4 | 40 | 30 | 6 |
| | Left frontal orbital cortex | 4.6 | -28 | 26 | -4 |
| | Left inferior parietal lobe | 4.9 | -48 | -44 | 42 |
| False > True | | | | | |
| | Posterior cingulate gyrus | 4.1 | -2 | -32 | 48 |

*Note.* True: true memory. False: false memory. NA: not applicable.

Supplementary Table 12. Brain regions showing differences in item-specific neural pattern similarity between true memory and correct control for OP (between original-event and post-event stages), OM (between original-event and memory-test stages), and PM (between post-event and memory-test stages) in Exp. 2 (thresholded at Z > 2.3).

| Contrast | Region | Z | x | y | z |
|---|---|---|---|---|---|
| Neural pattern similarity between original-event and post-event stages (OP: item-specific representation) | | | | | |
| True > Correct | | | | | |
| | Left inferior frontal gyrus | 3.4 | -52 | 32 | 14 |
| | Right inferior frontal gyrus | 4.2 | 54 | 12 | 22 |
| | Medial prefrontal cortex | 4.1 | -6 | 58 | -2 |
| | Left precentral gyrus | 4.6 | -54 | 8 | 30 |
| | Left inferior parietal lobe | 5.2 | -62 | -50 | 30 |
| | Left inferior temporal gyrus | 4.0 | -40 | -42 | -16 |
| | Left lateral occipital cortex | 4.5 | -34 | -84 | -12 |
| | Right lateral occipital cortex | 5.0 | 28 | -92 | -14 |
| Correct > True | | | | | |
| | Right superior parietal lobe | 4.2 | 28 | -46 | 62 |
| Neural pattern similarity between original-event and memory-test stages (OM: item-specific representation) | | | | | |
| True > Correct | | | | | |
| | Left inferior frontal gyrus | 5.6 | -50 | 34 | 12 |
| | Right middle frontal gyrus | 4.3 | 48 | 30 | 24 |
| | Left precentral gyrus | 4.5 | -40 | 6 | 24 |
| | Left inferior parietal lobe | 4.6 | -48 | -46 | 40 |
| | Left inferior temporal gyrus | 5.4 | -46 | -48 | -12 |
| | Left lateral occipital cortex | 6.1 | -32 | -92 | 20 |
| | Right lateral occipital cortex | 5.0 | 38 | -84 | -16 |
| Correct > True | | | | | |
| | NA | | | | |
| Neural pattern similarity between post-event and memory-test stages (PM: item-specific representation) | | | | | |
| True > Correct | | | | | |
| | Left inferior frontal gyrus | 4.7 | -44 | 20 | 8 |
| | Right inferior frontal gyrus | 5.5 | 44 | 30 | 12 |
| | Paracingulate gyrus | 5.8 | -10 | 20 | 48 |
| | Left inferior parietal lobe | 6.2 | -50 | -42 | 42 |
| | Right inferior parietal lobe | 4.0 | 56 | -52 | 34 |
| | Right middle temporal gyrus | 5.4 | 62 | -52 | -4 |
| | Left inferior temporal gyrus | 4.8 | -52 | -52 | -12 |
| | Left lateral occipital cortex | 5.7 | -30 | -72 | 26 |
| Correct > True | | | | | |
| | NA | | | | |

*Note.* NA: not applicable.

Supplementary Table 13. Brain regions showing differences in item-specific neural pattern similarity between correct and incorrect control for OP (between original-event and post-event stages), OM (between original-event and memory-test stages), and PM (between post-event and memory-test stages) in Exp. 2 (thresholded at Z > 2.3).

| Contrast | Region | Z | x | y | z |
|---|---|---|---|---|---|
| Neural pattern similarity between original-event and post-event stages (OP: item-specific representation) | | | | | |
| Correct > Incorrect | | | | | |
| | Left middle frontal gyrus | 4.0 | -48 | 12 | 47 |
| | Left superior frontal gyrus | 4.8 | -12 | 40 | 54 |
| | Medial prefrontal cortex | 3.9 | 4 | 54 | 12 |
| | Left superior parietal lobe | 4.4 | -36 | -78 | 38 |
| | Right inferior parietal lobe | 4.9 | 58 | -50 | 32 |
| | Right postcentral gyrus | 4.0 | 34 | -32 | 44 |
| | Precuneus | 4.3 | 0 | -60 | 36 |
| | Left inferior temporal gyrus | 4.0 | -50 | -52 | -16 |
| Incorrect > Correct | | | | | |
| | NA | | | | |
| Neural pattern similarity between original-event and memory-test stages (OM: item-specific representation) | | | | | |
| Correct > Incorrect | | | | | |
| | Right frontal pole | 3.9 | 54 | 40 | 18 |
| | Left precentral gyrus | 3.9 | -50 | 0 | 30 |
| | Left superior parietal lobe | 4.5 | -24 | -60 | 56 |
| | Right superior parietal lobe | 4.0 | 38 | -60 | 48 |
| | Left inferior temporal gyrus | 4.7 | -50 | -50 | -12 |
| | Right inferior temporal gyrus | 3.7 | 54 | -56 | -18 |
| Incorrect > Correct | | | | | |
| | NA | | | | |
| Neural pattern similarity between post-event and memory-test stages (PM: item-specific representation) | | | | | |
| Correct > Incorrect | | | | | |
| | Left precentral gyrus | 4.7 | -44 | 2 | 32 |
| | Left middle frontal gyrus | 3.6 | -28 | 28 | 48 |
| | Right middle frontal gyrus | 4.4 | 30 | 34 | 48 |
| | Medial prefrontal cortex | 4.1 | -4 | 54 | -8 |
| | Left superior parietal lobe | 5.1 | -18 | -78 | 52 |
| | Left inferior temporal gyrus | 3.8 | -58 | -48 | -14 |
| | Precuneus | 4.3 | -2 | -60 | 48 |
| Incorrect > Correct | | | | | |
| | NA | | | | |

*Note.* NA: not applicable.

Supplementary Table 14. Brain regions showing univariate activation differences between true and false memories during the original-event, post-event, and memory-test stages in Exp. 2 (thresholded at Z > 2.3).

| Contrast | Region | Z | x | y | z |
|---|---|---|---|---|---|
| Univariate activations during the original-event stage | | | | | |
| True > False | | | | | |
| | Left inferior frontal gyrus | 4.0 | -52 | 28 | -2 |
| | Right inferior frontal gyrus | 4.4 | 58 | 22 | 24 |
| | Right middle frontal gyrus | 3.4 | 36 | 4 | 52 |
| | Medial prefrontal cortex | 3.6 | -8 | 20 | 60 |
| | Left fusiform gyrus | 6.2 | -44 | -66 | -12 |
| | Right fusiform gyrus | 5.8 | 46 | -64 | -18 |
| | Left lateral occipital cortex | 4.6 | -30 | -58 | 46 |
| | Right lateral occipital cortex | 4.6 | 34 | -72 | 30 |
| False > True | | | | | |
| | Right frontal pole | 4.4 | 28 | 44 | 42 |
| | Medial prefrontal cortex | 3.5 | 6 | 40 | 12 |
| | Posterior cingulate gyrus | 4.4 | 6 | -26 | -26 |
| | Precuneus | 5.8 | -8 | -68 | 32 |
| Univariate activations during the post-event stage | | | | | |
| True > False | | | | | |
| | Left frontal pole | 5.5 | -46 | 41 | 8 |
| | Right frontal pole | 5.0 | 42 | 46 | -16 |
| | Left orbital frontal cortex | 6.4 | -30 | 28 | -2 |
| | Right orbital frontal cortex | 6.5 | 34 | 24 | -4 |
| | Medial prefrontal cortex | 6.8 | -4 | 38 | 36 |
| | Left inferior parietal lobe | 6.2 | -50 | -46 | 46 |
| | Right inferior parietal lobe | 6.5 | 46 | -54 | 46 |
| | Precuneus | 4.1 | -8 | -70 | 40 |
| | Posterior cingulate gyrus | 4.6 | 0 | -34 | 38 |
| | Left middle temporal gyrus | 4.0 | -66 | -26 | -14 |
| | Right middle temporal gyrus | 5.7 | 60 | -38 | -12 |
| False > True | | | | | |
| | Left superior parietal lobe | 4.2 | -28 | -44 | 54 |
| | Right superior parietal lobe | 4.5 | 16 | -52 | 72 |
| | Left superior temporal gyrus | 4.2 | -60 | 2 | -12 |
| | Right superior temporal gyrus | 4.4 | 56 | -2 | -12 |
| | Left middle temporal gyrus | 4.8 | -47 | -51 | 6 |
| | Right middle temporal gyrus | 4.5 | 46 | -50 | 12 |
| | Right parahippocampal gyrus | 4.0 | 27 | -27 | -18 |

| | | | | | | |
|---|---|---|---|---|---|---|
| Univariate activations during the memory test | | | | | | |
| | True > False | | | | | |
| | | Left frontal pole | 5.2 | -38 | 58 | -8 |
| | | Right frontal pole | 6.3 | 24 | 58 | 24 |
| | | Left orbital frontal cortex | 5.2 | -32 | 18 | -12 |
| | | Right inferior frontal gyrus | 5.0 | 54 | 22 | 0 |
| | | Medial prefrontal cortex | 7.0 | -8 | 46 | 4 |
| | | Left inferior parietal lobe | 6.0 | -36 | -48 | 38 |
| | | Right inferior parietal lobe | 6.3 | 54 | -48 | 54 |
| | | Cingulate gyrus | 5.6 | 2 | -6 | 30 |
| | | Left inferior temporal gyrus | 5.6 | -56 | -28 | -12 |
| | | Right middle temporal gyrus | 6.0 | 66 | -18 | -14 |
| | False > True | | | | | |
| | | NA | | | | |

*Note.* NA: not applicable.

Supplementary Table 15. Brain regions showing univariate activation differences between true memory and correct control during the original-event, post-event, and memory-test stages in Exp. 2 (thresholded at Z > 2.3).

| Contrast | Region | Z | x | y | z |
|---|---|---|---|---|---|
| Univariate activations during the original-event stage | | | | | |
| True > Correct | | | | | |
| | Right frontal pole | 3.8 | 38 | 38 | -10 |
| | Left inferior frontal gyrus | 6.7 | -48 | 10 | 30 |
| | Right inferior frontal gyrus | 6.2 | 46 | 12 | 28 |
| | Medial prefrontal cortex | 4.8 | -2 | 42 | -20 |
| | Left middle temporal gyrus | 5.5 | -64 | -46 | -4 |
| | Right middle temporal gyrus | 5.8 | 62 | -44 | -10 |
| | Left fusiform cortex | 6.7 | -32 | -34 | -26 |
| | Right fusiform cortex | 5.7 | 36 | -38 | -24 |
| | Left lateral occipital cortex | 5.5 | -40 | -86 | -14 |
| | Right lateral occipital cortex | 7.6 | 36 | -90 | -10 |
| Correct > True | | | | | |
| | Right frontal pole | 4.0 | 22 | 55 | -2 |
| | Right inferior parietal lobe | 3.9 | 50 | -40 | 14 |
| | Cuneal cortex | 6.1 | 8 | -70 | 24 |
| | Left occipital pole | 5.4 | -8 | -98 | 8 |
| | Right occipital pole | 6.1 | 16 | -96 | 8 |
| Univariate activations during the post-event stage | | | | | |
| True > Correct | | | | | |
| | Left frontal pole | 6.2 | -44 | 40 | 8 |
| | Right frontal pole | 4.7 | 44 | 48 | -16 |
| | Left insula | 7.1 | -30 | 22 | -6 |
| | Right insula | 7.7 | 32 | 24 | -4 |
| | Paracingulate gyrus | 7.1 | -4 | 24 | 46 |
| | Left inferior parietal lobe | 5.3 | -50 | -54 | 52 |
| | Right inferior parietal lobe | 4.7 | 50 | -50 | 44 |
| | Left middle temporal gyrus | 4.5 | -66 | -28 | -14 |
| | Right inferior temporal gyrus | 5.5 | 60 | -38 | -14 |
| Correct > True | | | | | |
| | Medial prefrontal cortex | 3.3 | 6 | 60 | 34 |
| | Precuneus | 5.4 | 6 | -70 | 56 |
| | Left superior temporal gyrus | 4.0 | -54 | 4 | -16 |
| | Right superior temporal gyrus | 3.8 | 62 | 6 | -10 |
| | Left middle temporal gyrus | 5.4 | -50 | -54 | 2 |
| | Right middle temporal gyrus | 5.7 | 56 | -62 | 12 |

| | | | | | |
|---|---|---|---|---|---|
| Univariate activations during the memory test | | | | | |
| True > Correct | | | | | |
| Left frontal pole | 7.1 | -40 | 56 | -8 |
| Right frontal pole | 6.7 | 38 | 54 | -2 |
| Left middle frontal gyrus | 4.5 | -46 | 26 | 42 |
| Right middle frontal gyrus | 5.9 | 42 | 30 | 46 |
| Right insula | 5.4 | 38 | 20 | -2 |
| Posterior cingulate gyrus | 5.7 | -2 | -30 | 38 |
| Left inferior parietal lobe | 6.5 | -48 | -50 | 48 |
| Right inferior parietal lobe | 6.7 | 40 | -56 | 50 |
| Left middle temporal gyrus | 5.0 | -64 | -24 | -10 |
| Right middle temporal gyrus | 5.5 | 70 | -22 | -10 |
| Correct > True | | | | |
| Left central opercular cortex | 5.3 | -50 | -6 | 14 |
| Precuneus | 5.8 | -2 | -58 | 44 |
| Left middle temporal gyrus | 4.7 | -48 | -64 | 12 |
| Right middle temporal gyrus | 5.3 | 40 | -60 | 16 |
| Left superior temporal gyrus | 3.8 | -50 | -6 | -16 |
| Right superior temporal gyrus | 3.9 | 48 | -6 | -18 |
| Right cuneal cortex | 4.0 | 4 | -82 | 18 |

Supplementary Table 16. Brain regions showing univariate activation differences between correct and incorrect control during the original-event, post-event, and memory-test stages in Exp. 2 (thresholded at Z > 2.3).

| Contrast | Region | Z | x | y | z |
|---|---|---|---|---|---|
| Univariate activations during the original-event stage | | | | | |
| Correct > Incorrect | | | | | |
| | Left superior parietal lobe | 4.4 | -30 | -54 | 44 |
| | Right superior parietal lobe | 4.1 | 26 | -54 | 44 |
| | Left fusiform gyrus | 4.6 | -44 | -84 | -14 |
| | Right fusiform gyrus | 3.2 | 12 | -74 | 2 |
| Incorrect > Correct | | | | | |
| | Medial prefrontal cortex | 4.0 | 4 | 38 | 12 |
| | Paracingulate gyrus | 3.6 | -2 | 28 | 30 |
| | Precuneus | 4.0 | -6 | -70 | 36 |
| | Posterior cingulate gyrus | 4.1 | 2 | -22 | 30 |
| Univariate activations during the post-event stage | | | | | |
| Correct > Incorrect | | | | | |
| | Left middle frontal gyrus | 5.5 | -44 | 8 | 34 |
| | Right middle frontal gyrus | 4.4 | 42 | 12 | 34 |
| | Left superior parietal lobe | 4.0 | -32 | -70 | 60 |
| | Left superior temporal gyrus | 4.7 | -54 | -2 | -14 |
| | Right fusiform gyrus | 4.3 | 38 | -46 | -20 |
| Incorrect > Correct | | | | | |
| | Left frontal pole | 5.3 | -26 | 46 | -14 |
| | Right frontal pole | 4.9 | -36 | 46 | 20 |
| | Right superior frontal gyrus | 4.9 | 18 | 18 | 64 |
| | Left insula | 4.1 | -40 | 14 | 0 |
| | Medial prefrontal cortex | 5.1 | 0 | 32 | 26 |
| | Left inferior parietal lobe | 3.7 | -62 | -48 | 44 |
| | Right inferior parietal lobe | 4.7 | 62 | -40 | 44 |
| | Posterior cingulate gyrus | 5.3 | 6 | -32 | 48 |
| Univariate activations during the memory test | | | | | |
| Correct > Incorrect | | | | | |
| | Right frontal pole | 4.0 | 28 | 68 | 2 |
| | Left precentral gyrus | 4.5 | -60 | 0 | 10 |
| | Medial prefrontal cortex | 4.1 | -2 | 66 | 14 |
| | Left inferior parietal lobe | 5.6 | -46 | -62 | 56 |
| | Right inferior parietal lobe | 3.9 | 52 | -68 | 40 |
| | Posterior cingulate gyrus | 4.8 | 0 | -30 | 30 |
| | Left middle temporal gyrus | 4.6 | -68 | -30 | -10 |
| | Right middle temporal gyrus | 4.1 | 64 | -32 | -12 |
| | Right lingual gyrus | 4.0 | 8 | -84 | -6 |

| | | | | | |
|---|---|---|---|---|---|
| Incorrect > Correct | | | | | |
| | Right superior frontal gyrus | 4.2 | 22 | 14 | 58 |
| | Left precentral gyrus | 3.9 | -40 | 0 | 44 |
| | Paracingulate gyrus | 5.1 | -6 | 14 | 46 |
| | Precuneus | 4.0 | -12 | -56 | 50 |

Supplementary Table 17. Brain regions showing stronger informational connectivity between hippocampus and cortex for true memory than false memory in OM (neural pattern similarity between original-event and memory-test stages) and stronger informational connectivity between hippocampus and cortex for false memory than true memory in PM (neural pattern similarity between post-event and memory-test stages) in Exp. 2 (thresholded at Z > 1.7).

| Contrast | Region | Z | x | y | z |
|---|---|---|---|---|---|
| Informational connectivity between hippocampus and cortex in OM | | | | | |
| True > False | | | | | |
| | Right middle frontal gyrus | 3.3 | 42 | 18 | 22 |
| | Left supramarginal gyrus | 3.7 | -64 | -42 | 20 |
| | Left angular gyrus | 3.1 | -54 | -64 | 38 |
| | Right lingual gyrus | 4.0 | 8 | -72 | -4 |
| Informational connectivity between hippocampus and cortex in PM | | | | | |
| False > True | | | | | |
| | Precuneus (extending to the posterior cingulate gyrus) | 3.9 | -2 | -62 | 30 |
| | Left fusiform gyrus | 3.8 | -44 | -58 | -18 |

*Supplementary Methods*

Training before the formal experiment:

Before the participants began each of the three stages of the formal experiment, there was a corresponding training program. For the training of the original-event stage, there were 10 images of an event (i.e., an animated Pixar movie “Cloudy”) that was unrelated to the 8 events in the formal experiment. For the training of the post-event stage, there were 10 narrative sentences (one for each image), which described these images accurately. For the training of the memory test, 8 questions would be asked for eight of the ten original images. The procedure of training was the same as for the formal experiment. There was no feedback or warning. To minimize the influence of experimenters, the experimental instructions were recorded as videos which were shown to participants before each stage.

## Supplementary References


1. Baym, C. L. & Gonsalves, B. D. Comparison of neural activity that leads to true memories, false memories, and forgetting: An fMRI study of the misinformation effect. *Cogn. Affect. Behav. Neurosci.* **10**, 339-348 (2010).
2. Okado, Y. & Stark, C. E. Neural activity during encoding predicts false memories created by misinformation. *Learn. Mem.* **12**, 3-11 (2005).
3. Putnam, A. L., Sungkhasettee, V. W. & Roediger, H. L. When misinformation improves memory: The effects of recollecting change. *Psychol. Sci.* **28**, 36-46 (2017).
4. Stark, C. E., Okado, Y. & Loftus, E. F. Imaging the reconstruction of true and false memories using sensory reactivation and the misinformation paradigms. *Learn. Mem.* **17**, 485-488 (2010).
5. St Jacques, P. L., Olm, C. & Schacter, D. L. Neural mechanisms of reactivation-induced updating that enhance and distort memory. *Proc. Natl. Acad. Sci. U.S.A.* **110**, 19671-19678 (2013).
6. Edelson, M., Sharot, T., Dolan, R. J. & Dudai, Y. Following the crowd: Brain substrates of long-term memory conformity. *Science* **333**, 108-111 (2011).
7. Karanian, J. M. *et al.* Protecting memory from misinformation: Warnings modulate cortical reinstatement during memory retrieval. *Proc. Natl. Acad. Sci. U.S.A.* **117**, 22771-22779 (2020).
8. Meng, X., Rosenthal, R. & Rubin, D. B. Comparing correlated correlation

coefficients. *Psychol. Bull.* **111**, 172-175 (1992).

9. Diedenhofen, B. & Musch, J. cocor: A comprehensive solution for the statistical comparison of correlations. *PLoS One* **10**, e0121945 (2015).

10. Chadwick, M. J. *et al.* Semantic representations in the temporal pole predict false memories. *Proc. Natl. Acad. Sci. U.S.A.* **113**, 10180-10185 (2016).
